# Physical Exercise-Induced Myokines and Muscle-Adipose Tissue Crosstalk: A Review of Current Knowledge and the Implications for Health and Metabolic Diseases

**DOI:** 10.3389/fphys.2018.01307

**Published:** 2018-09-24

**Authors:** Luana G. Leal, Magno A. Lopes, Miguel L. Batista

**Affiliations:** ^1^Integrated Group of Biotechnology, Laboratory of Adipose Tissue Biology, University of Mogi das Cruzes, São Paulo, Brazil; ^2^Technological Research Group, University of Mogi das Cruzes, São Paulo, Brazil

**Keywords:** IL-6, irisin, browning, inflammation, immunometabolism, exercise-factor

## Abstract

Physical exercise has beneficial effects on metabolic diseases, and a combined therapeutic regimen of regular exercise and pharmaceutical treatment is often recommended for their clinical management. However, the mechanisms by which exercise produces these beneficial effects are not fully understood. Myokines, a group of skeletal muscle (SkM) derived peptides may play an important part in this process. Myokines are produced, expressed and released by muscle fibers under contraction and exert both local and pleiotropic effects. Myokines such as IL-6, IL-10, and IL-1ra released during physical exercise mediate its health benefits. Just as exercise seems to promote the myokine response, physical inactivity seems to impair it, and could be a mechanism to explain the association between sedentary behavior and many chronic diseases. Myokines help configure the immune-metabolic factor interface and the health promoting effects of physical exercise through the release of humoral factors capable of interacting with other tissues, mainly adipose tissue (AT). AT itself secretes proinflammatory cytokines (adipokines) as a result of physical inactivity and it is well recognized that AT inflammation can lead to the development of metabolic diseases, such as type 2 diabetes mellitus (T2DM) and atherosclerosis. On the other hand, the browning phenotype of AT has been suggested to be one of the mechanisms through which physical exercise improves body composition in overweight/obese individuals. Although, many cytokines are involved in the crosstalk between SkM and AT, in respect of these effects, it is IL-6, IL-15, irisin, and myostatin which seem to have the decisive role in this “conversation” between AT and SkM. This review article proposes to bring together the latest “state of the art” knowledge regarding Myokines and muscle-adipose tissue crosstalk. Furthermore, it is intended to particularly focus on the immune-metabolic changes from AT directly mediated by myokines.

## Introduction

### General characterization

**Figure 1:**
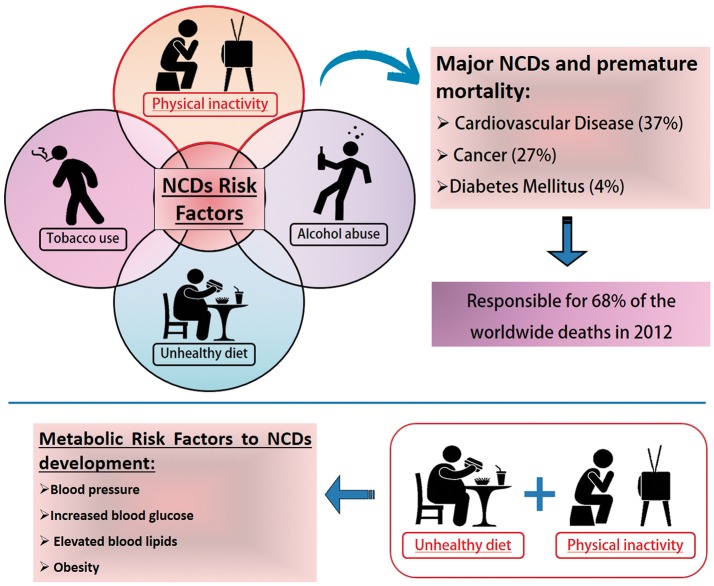
Noncommunicable Diseases (NCDs) Risk Factors. NCDs have become a major global public health problem. Tobacco and alcohol (misuse) use, unhealthy diet and physical inactivity represent important health risks. Physical inactivity is a leading cause of death worldwide. It increases the risk of major noncommunicable diseases (NCDs) such as coronary heart disease, type 2 diabetes and breast, and colon cancers, responsible in 2008 for an estimated five million deaths or about 9% of the total global premature mortality. According to the World Health Organization, poor life habits such as physical inactivity and unhealthy diets are the main indications for the development of metabolic disorders that increase the risk factors for the development of NCDs. Data available at: http://www.who.int/news-room/fact-sheets/detail/noncommunicable-diseases.

Physical inactivity is a global health problem, and recent data indicate that approximately one-third of the world's adult population is physically inactive. This means that these individuals do not perform the minimum 150 min a week of moderate to vigorous aerobic physical activity recommended by the World Health Organization (Ruiz-Casado et al., 2017). Physical inactivity is directly related to higher risk rates for major non-communicable diseases (NCDs). NCDs are a set of diseases of long duration and generally slow progression. The four main types of noncommunicable diseases are cardiovascular diseases (e.g., heart attacks and stroke), cancer, chronic respiratory diseases (e.g., chronic obstructive pulmonary disease and asthma) and type 2 diabetes mellitus (T2DM; WHO, 2014). In 2008, NCDs, such as coronary heart disease, T2DM and colon and breast cancers were responsible for about five million deaths or about 9% of total global premature mortality (Figure 1; Lee et al., 2012; Lobelo et al., 2014).

The benefits of physical exercise as a protective factor against NCDs have been documented since 450 BC, with Hippocrates, the “father of Western medicine,” stating that “Walking is man's best medicine” and “If there is a deficiency in food and exercise the body will fall sick” (Febbraio, 2017). Several biological mechanisms may be responsible for the reduction of risk factors for chronic diseases and premature death associated with physical exercise (Warburton et al., 2006) and there is currently a consensus in the literature that regular physical exercise is directly related to: (1) improvements in body composition, including reduced abdominal adiposity and body weight (Thomas et al., 2013; Smith, 2018); (2) improved lipoprotein protein profiles, through reduced triglyceride and low-density lipoproteins [LDL] levels, and increased high density lipoprotein [HDL] levels (Warburton et al., 2006; Mitsui et al., 2012); (3) greater efficiency in glucose homeostasis and insulin sensitivity (Tan et al., 2018); (4) reduced blood pressure (Sharman et al., 2015; Imazu et al., 2017); (5) reduced systemic inflammation (Lira et al., 2009; Rosa Neto et al., 2009; Batista et al., 2010) and (6) increased cardiac function (Barauna et al., 2005; Laterza et al., 2007; Batista et al., 2008).

Unhealthy lifestyles comprising unhealthy diet combined with lack of physical activity may result in increased blood pressure, increased blood glucose, elevated blood lipids and obesity. These are called metabolic risk factors that lead to cardiovascular disease, the leading NCDs in terms of premature deaths (GBD 2015 Risk Factors Collaborators, 2016). There are four metabolic markers referred to as risk factors for NCDs: (1) raised blood pressure; (2) overweight/obesity; (3) hyperglycemia and (4) hyperglicidemia. High blood pressure (19% of overall deaths) followed by overweight and obesity and increased blood glucose are related to the higher metabolic risk factors that culminate in death (GBD 2015 Risk Factors Collaborators, 2016). Obesity is considered a global epidemiological health problem and is associated with the development of numerous metabolic diseases, such as: insulin resistance (Martin et al., 2015), Type II Diabetes (Pulgaron and Delamater, 2014), atherosclerosis (Lovren et al., 2015), non-alcoholic hepatic steatosis (Li et al., 2014; Negrin et al., 2014), hypertension (Paley and Johnson, 2018) and coronary heart disease (Warburton et al., 2006; Figure 1).

Physical exercise is an important non-pharmacological treatment option not only as a preventive agent, but also in the treatment of numerous metabolic diseases, such as type II diabetes, hypertension, and cardiovascular disease (Nunan et al., 2013; Mahtani et al., 2015). The mechanisms involved in the benefits generated by physical exercise against metabolic diseases are related to the increase in total energy expenditure and consequent reduction in the accumulation of fat mass, including a reduction in total body mass (Lancaster and Febbraio, 2014). For decades, it has been known that there is a direct relationship between obesity and inflammation and the development of NCDs (Cani and Jordan, 2018). Numerous studies have sought to elucidate which components of the immune system, as well as which molecules from local inflammation, could be related to the development of NCDs (Lancaster and Febbraio, 2014). In obesity, the immune cell profile in AT is substantially altered, for example, there is an accumulation of proinflammatory macrophages, neutrophils, and CD8+ T lymphocytes, while regulatory and eosinophilic T cells are substantially reduced. This process contributes to inflammation that culminates in the development of systemic insulin resistance (Osborn and Olefsky, 2012; Mraz and Haluzik, 2014).

Considering the role of physical exercise as a direct modulating agent of the benefits related to the general improvement of physiological variables involved in NCDs in recent years, several studies have characterized the role of skeletal striated muscle in this context (Pedersen and Febbraio, 2012; Pedersen, 2013). It is well established that SkM constitutes a key organ for the oxidation of lipid, acting as an elaborate energy production and consumption system that influences the whole body's energy metabolism (Iizuka et al., 2014). SkM tissue is composed of several cell types including muscle fibers, stem cells, fibroblasts, pericytes, adipocytes, motor neurons, and connective tissue. In addition, the muscular satellite cells play an important role in the cellular signaling process with neighboring cells, thus being a bioactive secretory factor (Sheehan et al., 2000). For almost half a century, researchers have hypothesized that musculoskeletal cells have a humoral factor, which is activated by increased demand for glucose during muscle contractions (Nielsen and Pedersen, 2008).

The concept of SkM as an immunogenic producer/secretor of cytokines, which exert an endocrine function, was proposed by Pedersen (2013), who proposed that such cytokines and other peptides should be classified as myokines (Pedersen, 2013). The discovery of the role of SkM as an endocrine organ expanded the knowledge of how the nervous, endocrine and immune systems contribute synergistically to the maintenance of body homeostasis (Febbraio and Pedersen, 2005; Karstoft and Pedersen, 2016). Myokines are an important local agent, having effects on metabolism, angiogenesis and muscle growth, as well as being capable of systemically acting on other organs and systems (Febbraio and Pedersen, 2005). A tissue of great metabolic importance that has been the subject of studies on crosstalk with SkM is adipose tissue (AT). AT is widely known, not only for its ability to store energy, but also for its important endocrine component. AT in mammals consists of at least two different functional types: white and brown (Rosen and Spiegelman, 2006). White adipose tissue (WAT) is the main site of energy storage and release of hormones and cytokines that modulate whole body metabolism and insulin resistance, whereas brown adipose tissue (BAT) is important both for energy expenditure and the release of energy as heat. This thermogenic effect is mediated by the expression of decoupling protein-1 (UCP1; Cypess et al., 2009).

Both AT and SkM are significantly affected by exercise. One of the adaptations of these tissues to exercise is the secretion of molecules capable of modulating not only local metabolism but also systemic metabolism (pleiotropic). These secreted molecules can act in an endocrine manner to facilitate crosstalk between these tissues (tissue-to-tissue) and thus work together to improve overall metabolic health. In this review, we sought to produce an update on the state of knowledge in relation to the potential of physical exercise as inducer of myokines capable of modulating AT, as well as the impact of this crosstalk on health maintenance and promotion.

### Physical exercise as an inducer of an anti-inflammatory response

In recent years, several well-designed studies have demonstrated that performing acute aerobic exercise is an important mediator of systemic anti-inflammatory response (Petersen and Pedersen, 2005; Pedersen and Fischer, 2007; Pedersen et al., 2007). Muscle contraction induced by physical exercise results in increased gene expression and secretion of interleukin-6 (IL-6) by skeletal myocyte. Consequently, depending on the exercise variables (volume, intensity, density), there is an increase in IL-6 plasma levels (Steensberg et al., 2003). Following acute aerobic exercise, there is an increase in the cytokines interleukin-10 (IL-10), interleukin-1 receptor antagonist (IL1-ra), and the soluble receptors of the tumor necrosis factor I and II (TNF I and II). This set of changes is characterized as an “anti-inflammatory effect” (Petersen and Pedersen, 2005).

Given this condition, the hypothesis that has been proposed is that the regular practice of physical exercise, organized in a exercise training program (chronic effect), exerts an anti-inflammatory effect induced by the sessions (acute effect), which leads to improved protection against chronic inflammatory situations, levels of proinflammatory cytokines and C-reactive protein (Petersen and Pedersen, 2005; Fischer, 2006; Lira et al., 2009; Batista et al., 2010). However, the possible mechanisms modulating this “beneficial” effect are not well established.

In general, this anti-inflammatory effect has been shown to be more evident in some pathological conditions, such as atherosclerosis, type II diabetes, obesity and heart failure (HF), especially those presenting a two- to three-fold systemic increase in the levels of proinflammatory cytokines and C-reactive protein (Fischer et al., 2007). In addition, this cytokine production profile that occurs during and immediately after physical exercise is dependent on several factors, such as; population (sedentary, presence of diseases, etc.), the intensity or duration of the physical exercise, glucose availability and sample collection time (Flynn et al., 2007).

In addition to this, several cross-sectional studies have shown that there is a slight increase in the plasma levels of proinflammatory molecules under various conditions, such as physical inactivity (sedentary; Petersen and Pedersen, 2005; Flynn et al., 2007; Pedersen, 2007; Pedersen et al., 2007) in elderly people (Bruunsgaard et al., 2003), as well as in patients with diseases such as intermittent claudication (Tisi et al., 1997), T2DM (Boule et al., 2001), and atherosclerosis, among others. Under these conditions, the term “chronic low-grade systemic inflammation” has been used to characterize a 2- to 3-fold increase in plasma levels of TNF-α, IL-1α, IL-6, IL-1ra, sTNFR1 and 2, and C-reactive protein (CRP), inflammatory markers noted as important both in the development and the progression of these inflammatory processes. Despite the evident correlation between exercise and anti-inflammatory effects, little information has been produced so far to explain the possible mechanisms responsible for the relationship between physical training and the reduction in these markers (Pedersen and Fischer, 2007). In these disease conditions, the origin of this systemic alteration is not well characterized, however, it has been proposed that WAT and peripheral blood mononuclear cells (especially lymphocytes) may be the main source of these cytokines (Steensberg et al., 2003; Fischer, 2006; Pedersen and Fischer, 2007).

#### Chronic effect of exercise: physical training and neuroimmune-metabolic response

In general, exercise training programs are usually characterized by repetitive phases of overload, overreaching, maintenance of overtraining and recovery (Steinacker et al., 2004). The overload phases are characterized by a difference between the total amount of the overload (volume x intensity x density) and the recovery time between the exercise training sessions. Recovery between exercise sessions is necessary to allow recuperation, and over time, an improvement in exercise performance, metabolism and homeostasis.

On the other hand, if the recovery time is insufficient a chronic state of changes (molecular, biochemical and regulatory) can be produced that compromise well-being, increase the incidence of illness and decrease physical performance. The balance between exercise and recovery determines the positive or “beneficial” outcomes or adaptations of a given period of exercise training (Lehmann et al., 1993b; Steinacker et al., 2004).

Lehmann et al. proposed a biphasic model of response to training overload (Lehmann et al., 1993a) involving predominantly: 1-peripheral mechanisms in the early stages of overload in the organism, and 2-central mechanisms in the more pronounced and lasting phases of the overload period, with the hypothalamus as a central integrator of all afferent signaling to the brain and having an important role in regulating central responses to stress and physical training (Steinacker et al., 2004). These interactions involve afferent information from the autonomic nervous system, direct metabolic effects, hormones, cytokines and also information from superior brain centers, demonstrating a complex interaction involving bi-directional communication between the neuroendocrine and immune systems (Spinedi and Gaillard, 1998).

Researchers have examined the interaction of various systems in relation to the stimulus provided by physical exercise (Pedersen et al., 2007; Ruiz-Casado et al., 2017; Ishiuchi et al., 2018). Many researchers have sought to examine the role of the SkM themselves, particularly peripheral factors probably originating from successive muscle contractions during exercise that mediate changes in the tissue itself as well as having a systemic effect on other organs such as the liver and AT.

### Adipose tissue

Currently, AT is characterized as an important endocrine organ present in the body, related both to the expression of various cytokines and to the regulation and coordination of energy homeostasis, blood pressure, immune function, angiogenesis, mechanical shock protection, and thermogenic function (Prins, 2002; Trayhurn and Wood, 2004). The cytokines that are produced in the AT are called adipokines, molecules which have properties that allow them to generate autocrine and paracrine effects, or even a systemic effect, thanks to their endocrine characteristic, being able to reach distant tissues and have metabolic consequences in the whole organism (Kershaw and Flier, 2004).

Histologically, AT is characterized as connective tissue with special properties (Sheehan et al., 2000). In anatomical terms, two different specialized types of AT with different origins and functions, known as WAT and BAT, are described (Tsoli et al., 2012). WAT can be found in different regions in the body, being responsible mainly for the storing excess energy inside its cells in the form of triacylglycerol (TG). This stored energy is used when there is a caloric deficit (Kajimura et al., 2015). The anatomic location of AT determines its metabolic identity and central functions. In humans, WAT is located in many different deposits, such as the intra-abdominal visceral deposit, whose deregulation is associated with greater risks for metabolic diseases; and the subcutaneous deposit that can transmit protective effects on energy homeostasis (Kusminski et al., 2016). On the other hand, BAT has a more limited distribution. In mice, large BAT deposits, including interscapular, axillary and cervical BAT, develop in the prenatal period and provide a source of heat to protect newborns against exposure to cold (Kajimura et al., 2015). In adult men, the highest concentration of BAT occurs in the interscapular region (Kajimura et al., 2015). Its cellular structure presents a great concentration of mitochondria, having as one of its main function the thermogenic control of the organism (Sidossis and Kajimura, 2015). This process occurs through a mitochondrial protein called uncoupling protein 1 (UCP-1), which has the property of dissipating energy in the form of heat, a process described as non-shivering thermogenesis. Accordingly, this process results in the maintenance of body temperature when exposed to extreme situations (e.g., low temperatures; Karamanlidis et al., 2007; Wu et al., 2013).

As regards cellularity, both WAT and BAT are composed of several cell types, such as fibroblasts, endothelial cells, immune cells, nerves, preadipocytes and adipocytes, where the latter changes shape and function between WAT and BAT. While WAT adipocytes are characterized by the presence of a single large lipid (unilocular) droplet, BAT maintains multiple small droplets (multilocular) within its adipocytes (Sidossis and Kajimura, 2015). Although WAT is morphologically and metabolically different from BAT, about 30 years ago it was seen that there were adipocytes in the WAT that had morphological similarities with the BAT adipocytes, which until then were believed to be the only ones that had the thermogenic property (Harms and Seale, 2013; Wu et al., 2013). These adipocytes, as in the BAT adipocytes, were multilocular, had a larger number of mitochondria and when stimulated by low temperatures received β3-adrenergic stimulation, expressed UCP-1, elevated cellular metabolism and finally dissipated energy in the form of heat (Wu et al., 2013). These white cells with characteristics of brown cells were named as beige cells or “brite-brown in white,” and the name given to this phenomenon of cellular differentiation was browning of WAT (Wu et al., 2012, 2013).

#### Metabolic diseases and systemic repercussions of AT

As already mentioned, both physical inactivity and NCDs, particularly those diseases characterized by metabolic diseases, present a clear morpho-functional dysfunction of AT. Taken together, these stressors, depending on their intensity and duration, result in the remodeling of the AT. This process is characterized by a set of changes, such as: modification of the adipocyte area (hypertrophy, hyperplasia or atrophy; Arner, 2000); disturbance in fatty acid turnover (lipolysis and lipogenesis; Arner et al., 2010); rearrangement of extra-cellular matrix components (Scherer, 2016); inflammation (Batista et al., 2012); modulation of the browning phenotype related to the thermogenic effect (Kir et al., 2014); among others. The end results of this process are both local (i.e., in tissue) and systemic (ectopic fat, insulin resistance, etc.) impairment of the normal physiological functions of this tissue.

Physical inactivity contributes to being overweight or obese and the development of chronic conditions, including cardiovascular diseases, T2DM, gallbladder disease and osteoarthritis (Greenwood et al., 2010). Obesity, the excessive accumulation of body fat (mainly due to an imbalance between energy intake and expenditure) is at the heart of all these problems. Excessive fat accumulation in the adipocytes can result in an imbalance in TG turnover, inflammation in the AT and a consequent increase in the secretion of a large number of proinflammatory factors, such as TNF-α, and others (Mraz and Haluzik, 2014). Excess AT and consequent dysfunction plays an important role in the pathophysiology of other metabolic diseases such as metabolic syndrome (Grundy, 2015).

On the other hand, people with a deficiency of AT (for example, lipodystrophy) can also be diagnosed with metabolic syndrome. This happens because in this case redistribution of fat is almost always directed to SkM which directly leads to insulin resistance (Grundy, 2015). In cancer cachexia, AT is affected early, prior to the establishment of the main signs and symptoms of the syndrome (Fouladiun et al., 2005). The breakdown in fatty acid turnover is the most well characterized physiological event, evidenced by the increase of lipolysis and changes in of lipogenesis (Henriques et al., 2017). Increases of cellular infiltrate, in particular macrophages (Batista et al., 2013), increased collagen and impairment of adipocyte turnover (Franco et al., 2017), has also been described. As a result, AT loses its TG storage capacity and, consequently, presents a reduction in the mass of this tissue. This scenario (AT mass loss) presents a negative correlation with cancer patient survival (Ebadi et al., 2017).

### Skeletal muscle as an immunogenic organ

#### The concept of myokines

**Figure 2:**
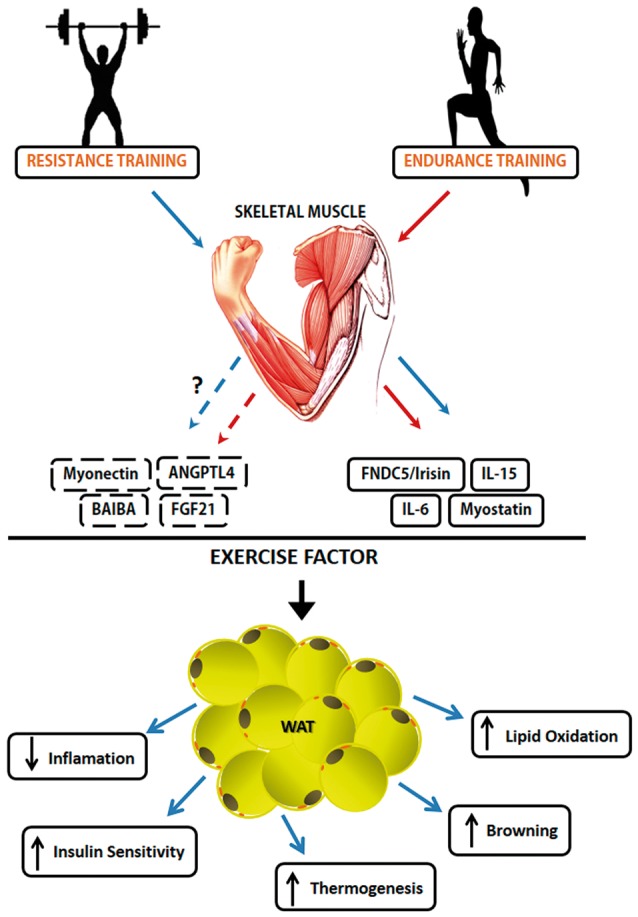
Myokines involved in induced-exercise muscle-adipose tissue crosstalk. The secretion and action of different types of Myokines is exercise type-dependent. Some Myokines like Irisin, Myostatin, IL-6 e IL-15 have been the subject of a number of published studies about their mechanisms. However, FGF21, Myonectin, ANGPTL4, and BAIBA need more studies about their role as exercise factor. Solid lines represent already known mechanisms, while broken lines represent the mechanisms that still have to be unraveled. The red line represents factors released during endurance training practice while the blue line represents factors released during resistance training practice. The Myokines released by exercise and their effects on White Adipose Tissue. Muscle contraction-induces a set of molecules that have an endocrine function. Muscle-adipose tissue crosstalk exerts functions such as reduction of adiposity, increase in thermogenesis due to increased adrenergic activity, increased markers of the browning phenomenon and increased lipolytic activity.

The SkM is a highly organized tissue at the micro and macroscopic level and is the body's main protein reserve (Hornberger, 2011; Bentzinger et al., 2013). SkM tissue is an important component related to quality of life, health, survival, and metabolic balance (Rodriguez et al., 2014; Salanova et al., 2014). It is a plastic tissue and continuously adaptable to various situations such as mechanical stimulation or disuse, leading to the condition of maintenance and/or muscular hypertrophy (increase of muscle mass) and atrophy (reduction of muscle mass), respectively. In addition, such tissue has a high capacity to alter its phenotype, depending on the mechanical load applied to it (Williamson et al., 2001). SkM tissue is also identified as an organ that synthesizes and secretes cytokines and other peptides, and these molecules have been given the name myokines (Pedersen et al., 2007). SkM is one of the largest organs of the human body, and in addition to its important functions such as providing locomotion, maintaining body temperature and metabolic homeostasis (Henningsen et al., 2010), the discovery that muscle contraction secretes proteins defined a new paradigm; SkM is a secretory organ, synthesizing and secreting myokines in response to muscle contraction. The products secreted by this tissue can influence the metabolism and function of SkM, in addition to other tissues and organs (Pedersen, 2013). Studies on the humoral component of SkM date back to the middle of the twentieth century, with a focus on the role of physical exercise as a modulator of glucose metabolism, with no conclusion about the mechanisms involved in this regulatory process (Goldstein, 1961). The ability of SkM to secrete myokines from muscle contraction was termed as “work stimulus,” “work factor,” or “exercise factor” (Figure 2; Pedersen, 2011). The concept of “exercise factor” was based on the fact that muscle contractions engage metabolic and physiological responses in other organs, and that these are not mediated by the nervous system (Pedersen et al., 2003). This concept was established after a study with electrical stimulation in paralyzed muscles, in which it was verified that the capacity of muscle contractions in modulating other tissues occurs through an independent pathway of activation of the nervous system (Kjaer et al., 1996). Thus, myokines began to be understood as a protective factor against disease and the effects of physical inactivity.

Table 1 provides a list of original articles that investigated the potential of exercise/physical training as an inducer of myokines and evidence of mechanisms or outcomes relating to the muscle-adipose tissue crosstalk.

**Table 1:** List of original papers that investigate proteins expressed and/or secreted by skeletal muscle which act on muscle-adipose tissue crosstalk.

**References**	**Experimental model**	**Training protocol**	**Myokines analyzed**	**Main results**	**Evidence of muscle-adipose tissue crosstalk**
Norheim et al., 2014	Healthy and physically inactive men	Combined strength and endurance training for 12 weeks, including two endurance bicycle sessions (60 min−70% VO_2_máx) and two whole body strength training sessions (60 min) per week.	FNDC5/Irisin	↑ FNDC5 mRNA expression in skeletal muscle. ↑ Plasma concentration of irisin after 45 min ergometer cycling (~1.2-fold) and ↓ after 2 h rest.	There was little or no effect of long-term training on selected browning genes and no correlation between circulating irisin with UCP1 mRNA in subcutaneous adipose tissue.
Lee et al., 2014	Healthy adults and volunteers wearing hospital scrubs rested in beds	Acute session of exercise on cycloergometer following graded, stepwise cold exposure to maximal capacity (VO2max) and sub-maximal exercise test at 40% VO2max for 1 h.	Irisin	↑ Irisin in maximal exercise group compared to cold exposure.	*In vitro* experiments (BAT) provide mechanistic insight into *in vivo* observations. The results indicate that shivering-stimulated irisin, in concert with FGF21, phenotypically transforms white adipocytes to BAT-like cells to expand overall thermogenic capacity.
Miyamoto-Mikami et al., 2015	Healthy young subjects and healthy middle-aged/older adults	Cycling on a leg ergometer for 55 min, 3 days/week, for 8 weeks.	Irisin	↑ Serum irisin level in the middle-aged/older training group ↔Serum irisin level in the young training group	In the middle-aged/older training group, the endurance training-induced reduction in visceral adipose tissue area was negatively correlated with the change in serum irisin level
Kurdiova et al., 2014	Middle-aged sedentary men	Combined training performed 3 times per week during 3 months. Intensity was maintained at 70–85% of maximal heart rate and strength training Intensity was maintained at 70–85% of maximal heart rate.	FNDC5/Irisin	Exercise did not affect *Fndc5*/irisin	Was observed a down regulation of Fndc5/irisin in adipose tissue and circulation in T2D group.
Roca-Rivada et al., 2013	Lean and obese rats and obese men	Free access to the activity wheel for 1 or 3 weeks	FNDC5/Irisin	↑ FNDC5/irisin in muscle after 3 weeks of exercise ↑ FNDC5/irisin in adipose tissue after 1 week of exercise and ↓ after 3 weeks exercise	75% of irisin expression by skeletal muscle and 28% by AT. No correlation between expression of UCP1 in subcutaneous fat and circulating irisin
Pekkala et al., 2013	Untrained and trained healthy men	Acute low-intensity aerobic exercise was performed with bicycle ergometer for 1 h at a low intensity of 50% *V*O2max Single resistance exercise of 5 sets of 10 repetitions in leg press until failure. Heavy-intensity endurance exercise, twice a week and combined EE and RE training	FNDC5/irisin	↑*FNDC5* mRNA only in young men post-RE	No change in plasma level of irisin on adipose tissue. Irisin and FNDC5were not associated with glucose tolerance and being overweight, or with metabolic disturbances, respectively
Bueno et al., 2011	Wistar rats	1.5 h/day, 45 min at 9:00 AM and 45 min at 05:00 h PM, for 4 weeks.	Myostatin	↔ In mRNA myostatin between exercised and sedentary normal diet and HFD rats	↑ In mRNA myostatin in BAT of high-fat rats after swimming ↓ In mRNA myostatin in mesenteric AT of high-fat trained rats versus sedentary high fat diet rats
Hjorth et al., 2016	Sedentary men	12 weeks of two interval bicycle sessions (60 min, 45-minbicycle test at 70% of VO2max) and two full-body strength training sessions (60 min) per week	Myostatin	↓mRNA myostatin in skeletal muscle after acute and long-term exercise In cultured muscle cells myostatin promoted an insulin-independent increase in glucose uptake. ↑ Enhanced rate of glucose oxidation and lactate production in muscle cells incubated with myostatin	The expression of myostatin was correlated negatively with insulin sensitivity ↑ Myostatin mRNA in AT after 12 weeks of training and correlated positively with insulin sensitivity markers.
Macpherson et al., 2015	C57BL/6 mice	Mice ran for 120 min at 15 m/min, with an incline of 5%.	IL-6	↑ Expression and secretion of IL-6 and IL-10 in skeletal muscle in exercise HFD group	↑ On infiltrates cells in AT of HFD group ↓ F480 and CD11 in AT in exercise HFD group
Castellani et al., 2015	C57BL/6 mice	Mice ran for 2 h at 15 m/min at a 5% incline, during 4 weeks	IL-6	↔ No differences in plasma IL-6 between sedentary and trained mice	↑ In levels of il-6 mRNA epididymal AT with no increase in IL-1β and TNF-α ↑ In protein and mRNA levels of IL-6 receptor in epididymal AT

*BAT, Brown Adipose Tissue; AT, Adipose Tissue; EE, Endurance Exercise; RE, Resistance Exercise; T2D, Type 2 Diabetes; ↑, increase, ↓, decrease and ↔, no alterations*.

### Myokines that act on crosstalk between SkM and AT

As described above, myokines are released from SkM in response to exercise. Thus, following the characterization of irisin as a myokine capable of activating adipose tissue browning in rodents in response to exercise (Bostrom et al., 2012) the concept that factors secreted by SkM could signal to other organs has grown and developed. More recently, Lee et al. (2014) shown that irisin and FGF21, both induced by submaximal exercise, have a function in promoting the browning of adipose tissue to meet the increased demand for fat oxidation. Moreover, the characterization of these two molecules opened an avenue of possibilities in the search for new factors that will add to our understanding of muscle-adipose tissues crosstalk and the options for treatment (Figure 2). In this review article, the main proteins identified in the literature that are expressed/secreted by SkM and have a capacity to act on the crosstalk between muscle-adipose tissues will be explored.

#### Myostatin

Transforming growth factor beta superfamily (TGF-β) and related factors such as myokines are first candidate molecules identified in SkM, and reported to induce biological signals that regulate cell growth, regeneration, differentiation, transformation and death of SkM (Iizuka et al., 2014). Among them are: myostatin, activins or inhibins, follistatin and bone morphogenic proteins (BMP). Myostatin is a protein related to the control of muscle growth and body metabolism (Feng and Derynck, 2005).

Among the different proteins of the TGF-β family, myostatin (also known as GDF8) is a protein secreted during embryonic development and its function is to limit muscle growth in the physiological situation during development. However, it is known today that myostatin is also expressed and secreted even in adulthood (Argiles et al., 2012). Myostatin circulates in the blood in a latent form and when cleaved, it presents itself actively. Once activated, it has high affinity to ACTRIIB Activins receptors (de Caestecker, 2004). Once attached to its receptor, it triggers the activation of the Smad family transcription factors (Smad2 and Smad3). This activation in turn culminates in muscle atrophy, through the subsequent activation of the Forkhead Box family transcription factors, FOXO (1, 2, and 3) together with inhibition of the AKT/mTOR pathway (Braun and Gautel, 2011). The importance of myostatin as an atrophic component is highlighted in patients with HF and cancer, who present increased serum levels of this protein, which can result in cachexia, which can only be reversed by the deletion of the gene, as can be observed in *in vitro* assays and transgenic animal models (George et al., 2010; Heineke et al., 2010).

In addition to the ability to modulate the Smad, FOXO and AKT pathways, the interaction between myostatin and physical exercise also appears to occur through the transcription factor peroxisome proliferator-activated receptor gamma co-activator 1-alpha (PGC-1α) in muscle, which in turn is stimulated by exercise (Han et al., 2013). Studies have shown that inhibition of myostatin results in up-regulation of PGC-1α activity in the SkM, thus stimulating mitochondrial biogenesis. PGC-1α in turn binds to FOXO and inhibits its transcriptional activity (Lebrasseur et al., 2009). Another effect is an increase in AMPK activity in the muscle, which increases insulin sensitivity and responsiveness (Zhang et al., 2011).

However, myostatin is not exclusively secreted by SkM tissue, with studies indicating that AT also secretes this protein. Its importance in this tissue is related to the maintenance of adipose mass and greater sensitivity to insulin (Hamrick et al., 2006; Guo et al., 2009). Myostatin mRNA levels increase substantially in genetically-modified obese mice (leptin-deficient *ob/ob* mice) and also in those receiving a high fat diet (HFD), so both circulating and SkM myostatin levels are present in obese subjects when compared to non-obese subjects (Allen et al., 2008; Hittel et al., 2009). Transgenic mice that expressed non-functional myostatin (conditioned to SkM), presented not only increased muscle mass but also resistance to weight gain (fat mass) and insulin resistance even when receiving a high-fat diet (Zhao et al., 2005). On the other hand, knockout animals for myostatin presented not only an increase in muscle mass but also an improvement in composition due to a reduction of fat mass, which in turn was due to the presence of the browning phenotype in the WAT, mainly because of an increase in the expression of irisin (Shan et al., 2013).

However, there are few studies in the literature that investigate the direct role of myostatin as an “exercise factor” and its ability to modulate AT. Bueno et al. (2011), evaluated the expression of myostatin and its ACTRIIb receptor in both SkM and AT from obese and insulin resistant rats who practiced swimming training. No change was found in myostatin expression in the SkM of trained animals compared to the control group. However, in the BAT the expression of myostatin and ACTRIIb was increased in the trained obese animals compared to the sedentary obese, while in the mesenteric adipose tissue (meAT) they were reduced (Table 1). The authors suggest that these changes are due to the ability of myostatin to modulate energetic homeostasis in exercise and obesity, since no change was observed in non-obese animals (Bueno et al., 2011). A recent study investigated the relationship between myostatin, physical activity and dysglycaemia in men with or without dysglycaemia who underwent a 30–45 min cycle test before and after 12 weeks of combined training (Hjorth et al., 2016). They found that myostatin mRNA expression was reduced in SkM after acute exercise and was further reduced over 12 weeks of training, while myostatin expression in AT increased after 12 weeks of training and correlated positively with insulin sensitivity markers (Table 1). Analysis at cellular levels with recombinant myostatin showed increased glucose uptake in human SkM cells, suggesting a complex regulatory role of myostatin in SkM homeostasis.

However, these last two studies have not yet elucidated direct evidence on muscle-adipose tissue crosstalk, since modulation in myostatin levels from exercise was due to qPCR analysis from AT. It is clear that myostatin is modulated by exercise, but more work on the modulation of myostatin muscle levels and its direct action in the exercise state are still necessary.

#### Interleukins

Interleukins (IL) are a group of cytokines bound to the immune system that have the ability to develop several different cellular responses when bound to surface receptors. These molecules have around 30 different isoforms that act in a paracrine and autocrine way, being strongly linked to inflammatory pathologies (Brocker et al., 2010). They play an important role in inflammatory processes and can exert a pro or anti-inflammatory function (Batista et al., 2010). The main proinflammatory cytokines are IL-6 and IL-1β, while the main anti-inflammatory ones are IL-4, IL-10, and IL-13, which act to inhibit the expression of other proinflammatory proteins (Lira et al., 2009). Other ILs such as IL-4, IL-8, IL-7, and IL-15 are also released by muscle tissue (Pedersen, 2011).

##### Interleukin-6 (IL-6)

Interleukin-6 (IL-6) is an important IL that is at high levels after exercise and plays an important role in systemic inflammation (Pratesi et al., 2013). High levels of IL-6 are observed in situations where glycogen levels are low, as a response to metabolic demand, since higher levels of IL-6 are related to a higher lipolytic rate by activation of the AMPK pathway and/or PI3-kinase activating the oxidation of the fatty acids which leads to greater availability of energy supply from this energy source (Keller et al., 2001).

Some studies suggest that increased IL-6 during and after an acute exercise session might be related to the type of exercise performed. Higher plasma levels of IL-6 have been reported in some studies that use running as a model of physical exercise compared to those which use cycling (Nieman et al., 1998; Starkie et al., 2001). According to these studies, the running model leads to a greater release of IL-6 because of the greater muscle damage which occurs due to its eccentric component being larger compared to that of cycling. Other studies comparing the IL-6 response in these two exercise models did not show significant differences (Starkie et al., 2001).

Increased levels of IL-6 after exercise are followed by increased expression of the IL-1 receptor antagonist (IL-1ra) and IL-10, such adjustment chronically constitutes an anti-inflammatory component and a response to the increase in circulating IL-6 induced by exercise (Febbraio and Pedersen, 2002). Plasma concentrations of IL-6 may increase up to 100-fold after exercise. However, the increase in circulating IL-6 after exercise does not appear linearly over time, with a study finding an exponential acceleration in IL-6 secretion soon after exercise (Macdonald et al., 2003). In a recent study, in which the effects of voluntary running in tumor-bearing mice were evaluated, running animals showed a 29-fold increase in IL-6 levels in SkM, increased secretion of NK cells and a reduction in tumor volume, whereas trained animals receiving an IL-6 antagonist did not exhibit the same rate of tumor reduction as well as less infiltration of NK cells into the tumor (Pedersen et al., 2016). Such a finding confirms the importance of IL-6 as an “exercise factor” with the ability to modulate the immune system during tumor progression.

However, studies have shown that IL-6 can induce an anti-inflammatory environment, not only by inducing the production of anti-inflammatory cytokines, but also, under specific conditions, by inhibiting the production of TNF-α, as demonstrated in an *in vitro* study (Beyaert and Fiers, 1999) and in mice (Petersen and Pedersen, 2005). In humans, infusion of rhIL-6, an experimental procedure that mimics the increase in IL-6 levels induced by exercise, was able to inhibit the increase in endotoxin-induced TNF-α plasma levels (Starkie et al., 2003). In addition, evidence of an anti-inflammatory effect of IL-6 has shown to be related to a direct relation between muscle tissue and AT. This kind of relationship is characterized as crosstalk (Macpherson et al., 2015).

This study was conducted in C57bl/6 mice that received a high fat diet (HFD) and were exposed to running training (Table 1). At the end of the study, it was possible to observe that even without changes in adipose mass, higher levels of IL-6 expression in the SkM were responsible for the increase in IL-10 expression, together with a significant reduction in inflammatory infiltrates in AT (Macpherson et al., 2015).

As described previously, IL-10 can act on different cell types to induce suppression of the inflammatory response and it has been postulated that it is the main molecule responsible for the “orchestration” of inflammatory reactions, in particular those involving the activation of monocyte/macrophage cells. Therefore, in humans, when IL-10 is added to the culture medium of mononuclear cells and circulating neutrophils stimulated with lipopolysaccharide(LPS), the synthesis of proinflammatory cytokines (TNF-α, IL-1β, IL-6) is inhibited through post-transcriptional mechanisms, a direct consequence of a higher mRNA degradation rate of the corresponding genes (Christiansen et al., 2010).

##### Interleukin-15 (IL-15)

Another IL expressed by SkM with an important metabolic role is IL-15. It has been suggested to be an important modulator of body fat mass, which also actively participates in the innate immune response, playing an important role in the development and function of natural killer (NK) cells and lymphocytes (Quinn et al., 2013).

In a study by Nielsen et al. which analyzed the rate of secretion of IL-15 in SkM of individuals who practiced Resistance Training (RT), it was possible to confirm that 24 h after the last RT session a two-fold increase in IL-15 mRNA levels was observed, and that this response was also dependent on the type of muscle fiber (Nielsen et al., 2007). Studies indicate that this cytokine is the main agent in crosstalk between SkM tissue and AT (Pedersen et al., 2007). The relationship of IL-15 and AT can be observed in a translational study (Nielsen et al., 2008), which verified in humans an inverse relationship between IL-15 (mRNA) and AT mass indexes. In mice transfected with IL-15 overexpressing plasmid, they showed reductions in AT mass compared to the control group.

The concentration of circulating IL-15 was shown to be closely related to physical exercise, in addition to increasing lipid oxidation and gene expression of peroxisome proliferator-activated receptor delta (PPARδ) in rodents when they were treated with the cytokine (Quinn et al., 2013). In addition, IL-15 is directly related to muscle metabolism, or even diet-induced obesity and insulin sensitivity (Quinn et al., 2011, 2013).

Because it has an anti-inflammatory function, IL-15 inhibits the action of TNFα in the muscle during cachexia (Pajak et al., 2008). In addition, any deregulation of this cytokine can lead to several pathologies linked to autoimmune diseases such as rheumatoid arthritis and leukemia (Fehniger and Caligiuri, 2001). However, further studies are required to establish its full clinical importance.

Some previous studies demonstrated the link between IL-15 release, SkM, and NCDs. In an *in vitro* study, cells treated with IL-15 had increased expression of heavy chain myosin and induced anabolism in SkM cells without stimulation of precursor myogenic cells (Furmanczyk and Quinn, 2003). Also, the ability of IL-15 to induce hypertrophy in SkM cells has been demonstrated through the reduction of the rate of protein degradation during cachexia and sarcopenia, highlighting the therapeutic power of this cytokine (Quinn et al., 2002). The ability of IL-15 to act on muscle- adipose tissue crosstalk was investigated by the same group, showing IL-15 overexpression in relation to reduced adiposity. To test the hypothesis, transgenic animals that overexpress IL-15 (mRNA and protein) in SkM were used in two experimental conditions: overexpression of muscle-free IL-15 in the circulation and overexpression of muscle IL-15 without secretion into the circulation. Only the group that had the increased levels of IL-15 from the muscle in circulation had a reduction in adiposity due to the greater targeting of this substrate for energy generation (Quinn et al., 2009). However, no study has been able to provide evidence of the direct mechanism of IL-15 as an “exercise factor” in muscle-adipose tissue crosstalk.

#### FGF-21

Fibroblast growth factor 21 (FGF21) is an endocrine-member of the FGF family. The expression and circulating levels of FGF21 are dependent on factors such as nutritional status, diet, hormone levels, and activities of various transcriptional factors. FGF21 acts in the control of glucose and lipid homeostasis (Kharitonenkov et al., 2005). In general, FGF21 stimulates the uptake of glucose by adipocytes and inhibits the production of glucose in the liver. In addition, FGF21 appears to protect pancreatic β cells from cell death induced by glycotoxicity (Kharitonenkov et al., 2005, 2007). Hojman et al. found that FGF21 is expressed in both plasma and SkM after insulin stimulation in humans, and thus classified FGF21 as an insulin regulatory myokine (Hojman et al., 2009). However, studies that aimed to correlate FGF21 and its importance as “exercise factor” are still inconclusive. A study by Cuevas-Ramos et al. found an increase in serum FGF21 levels in runners after 2 weeks of training, but found no change in their gene expression in SkM (Cuevas-Ramos et al., 2012). However, another study analyzed the expression of FGF21 after an acute session of running exercise in both an experimental mice model and in humans and showed an increase in serum FGF21 levels (Kim et al., 2013b). However, this increase was not in SkM or AT but in the liver (Kim et al., 2013b). The authors, observed that this increase is in turn accompanied by increased hepatic peroxisome proliferator-activated receptor alpha (PPARα) expression, required for the activation of lipolysis and also of ATF4 (Kim et al., 2013b), a transcription factor identified in several stress response situations, including autophagy, mitochondrial stress, and amino acid deprivation (Kim et al., 2013a). In this respect, more studies are needed to confirm the importance of FGF21 as “exercise factor,” since studies on the systemic effects of this molecule focus on quantifying their plasma levels (Cuevas-Ramos et al., 2012; Lee et al., 2014), rather than on verifying their expression in the SkM during contraction.

#### Irisin

Irisin is considered the most promising myokine in the context of metabolism maintenance because it is secreted by SkM and has been suggested to mediate the effect of exercise on WAT metabolism (Aydin et al., 2014). Irisin, formerly called Iris, is a recently identified myokine, released into the circulation by SkM in an exercise-dependent manner. It is reported to have the ability to convert WAT to BAT, a phenomenon known as browning; a phenotype set of adaptations that results in increasing total energy expenditure (Bostrom et al., 2012).

**Figure 3:**
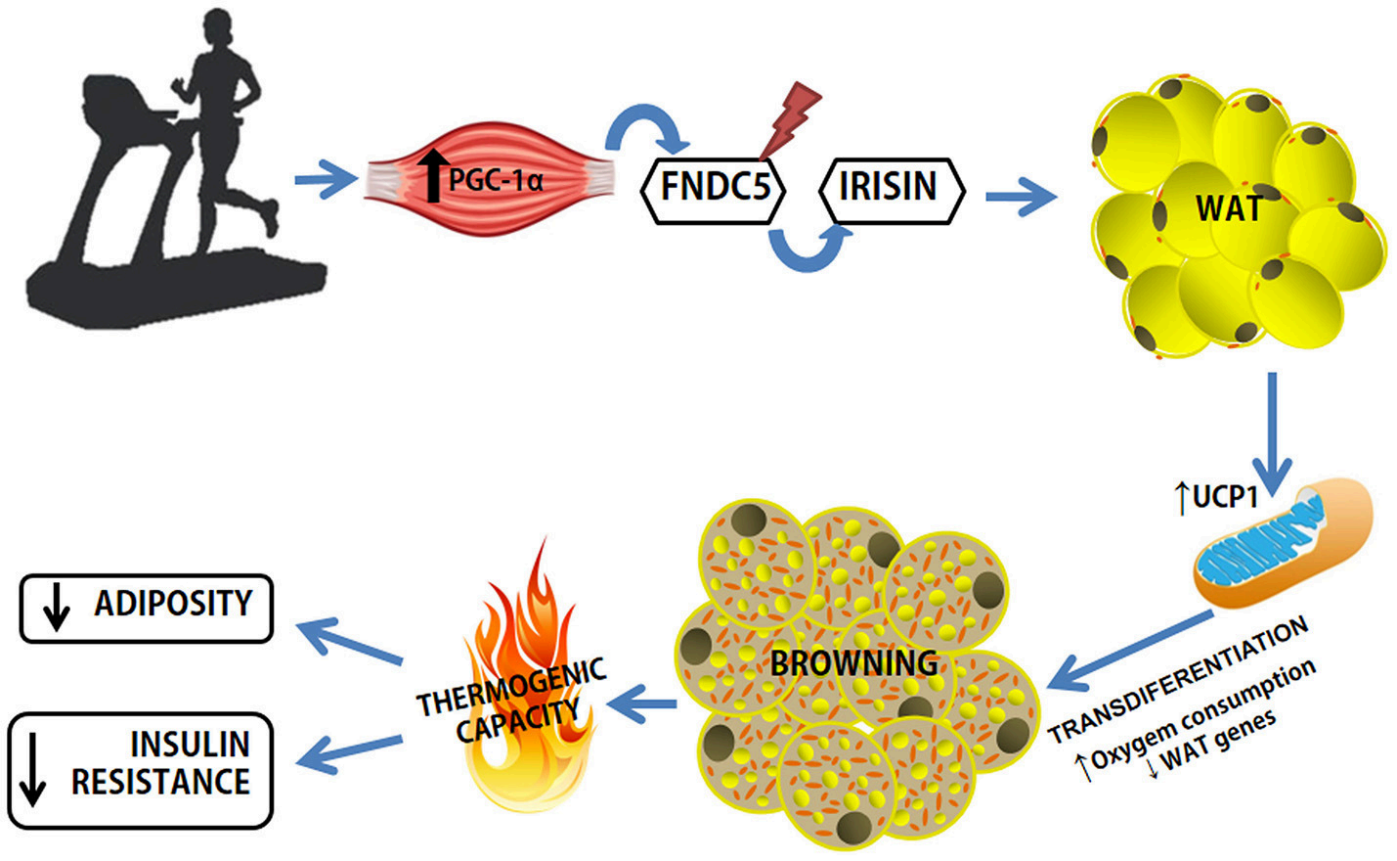
Exercise-induced browning in WAT. Increased expression of PGC-1a in muscle as a result of exercise causes increased expression of FNDC5, a type I membrane protein, which C-terminally cleaved and secreted as irisin into the circulation. Irisin binds to a receptor yet unknown in WAT adipocytes and leads to phenotypic modification. WAT, white adipose tissue; FNDC5, Fibronectin Type III Domain Containing 5; UCP1, Uncoupling Protein Type 1.

Irisin was discovered and characterized by Bostrom et al. (2012), in a study that sought to elucidate the relevance of the transcriptional coactivator PGC-1αin the control of obesity (Bostrom et al., 2012). First, an algorithm was used to predict candidate proteins produced by SkM that may mediate the browning process in AT, excluding mitochondrial target proteins (Figure 3). Five candidate proteins were found to be mediated by PGC-1α in the muscle: IL-15, Fndc5, VEGFβ, Lrg1, and TIMP4. However, only the irisin precursor FNDC5 was able to promote the differentiation of stromal vascular cells isolated from WAT from beige mice (browning phenomenon; Bostrom et al., 2012). Consequently, there was an emerging search for a deeper understanding of the mechanisms of action of irisin, whether they were local or systemic as well as its role as an “exercise factor” (Huh et al., 2012, 2014, 2015; Hecksteden et al., 2013; Moraes et al., 2013; Pekkala et al., 2013; Roca-Rivada et al., 2013; Aydin et al., 2014; Daskalopoulou et al., 2014; Pardo et al., 2014; Tsuchiya et al., 2014; Kim et al., 2015; Miyamoto-Mikami et al., 2015; Fagundo et al., 2016; Duft et al., 2017). However, most of the studies only analyzed plasma levels of FNDC5/irisin and did not assess its expression by SkM. A study aimed to verify the physiological variables effect of irisin in healthy groups of swimmers of different ages and explore the direct effects on muscle metabolism, and found: (1) higher irisin serum levels in the younger group compared to the older group (age effect); (2) serum irisin levels were elevated in the swimmer groups (training effect); (3) rates of plasma irisin were related to the intensity of exercise training, with the group that performed intermittent high-intensity exercise having higher levels; (4) Higher rates of irisin were related to the metabolism of glucose and lipids in SkM through AMPK phosphorylation; despite the differences in basal irisin levels, exercise-induced irisin secretion is independent of age or fitness level. Increased irisin can directly modulate muscle metabolism through 5' AMP-activated protein kinase (AMPK) activation (Huh et al., 2014).

Another study aimed to elucidate the association between endurance exercise and the impact of irisin induction on body fat in 25 healthy (± 21 years old) and elderly (± 68 years) individuals who practiced 8 weeks of cycloergometer training at 60–70% of VO2peak for 45min, 3x/week. The main results indicated an increase in serum irisin levels in the elderly group compared to the young group (age 21 ± 1 years) accompanied by a reduction in visceral adiposity which was negatively correlated with irisin levels (*r* = −0.54, *P* < 0.05; Miyamoto-Mikami et al., 2015). However, the irisin modulation seemed to be dependent on the modality and intensity of the exercise. In this respect, irisin secretion was observed in volunteers of different ages submitted to a protocol of aerobic exercise of moderate to maximum intensity (70–75% VO2max until exhaustion; Huh et al., 2014). In this study, it was possible to verify that intense exercise, until exhaustion, promoted higher irisin serum levels after exercise in the young group compared to the old group (age 67.9 ± 5.0). The same study also analyzed the secretion of irisin in swimming adolescents in groups that either practiced high or moderate intensity swimming. It was observed that there was a significant increase in serum irisin levels immediately and up to 1 h after an exercise session in the group that practiced high-intensity swimming compared to the moderate intensity group.

Norhein et al. argue that the regulatory effect of physical training on the expression of muscular FNDC5 is not clear. In this study, 26 physically inactive men (normoglycemic and diabetic) were submitted to a 12-week physical training program combining strength and endurance exercises (Table 1). Muscle biopsies were performed before and after the intervention and it was observed that muscle mRNA levels for both PGC-1α and FNDC5 were increased after exercise. However, circulating levels of irisin were only increased acutely and shortly after the exercise session (1.2-fold; Table 1). As far as the analysis of muscle-adipose tissue crosstalk was concerned, few effects were seen in AT browning: AT UCP1 mRNA levels were not directly correlated with FNDC5 levels in both AT and in SkM (Norheim et al., 2014). Pekkala et al. analyzed the effects of different short-term and long-term exercise protocols on the FNDC5 and PGC-1α of healthy trained and untrained men, who either did low intensity aerobic exercise, resistance training, high intensity exercise or combined endurance training, and assessed levels of muscle and irisin serum, as well as studying the associations of irisin and FNDC5 with health parameters (Pekkala et al., 2013). In this study, it was observed that muscle irisin levels were only increased in the young group that performed a single resistance training session, without any changes in irisin serum levels regardless of training. No association was found between levels of FNDC5 and health parameters (Table 1). The group also concluded that data on exercise and irisin modulation are questionable.

However, it is important to note that irisin is not a protein exclusively secreted by muscle tissue. The study presented by Arturo Roca-Rivada et al. was a pioneer in showing that WAT also secretes FNDC5, the precursor of irisin, indicating that it is not only a myokine but also an adipokine (Roca-Rivada et al., 2013). The study was based on gene and protein expression analyses by explant assay of subcutaneous and visceral AT from rats. He also found that both tissues secreted FNDC5/irisin under the stimulus of endurance training.

#### ANGPTL4

Angiopoietin-like protein (ANGPTL) is a group of proteins that can be secreted by the AT, SkM, intestinal, and liver cells and is responsible for several processes, among them energy metabolism, and TG turnover modulation in AT and the regulation of blood glucose levels (Ingerslev et al., 2017; Popova et al., 2018). Despite the name, ANGPTLs are not specific ligands for angiopoietin, but are named after the structural similarity with this protein (Cinkajzlova et al., 2018).

ANGPTL is regulated through peroxisome proliferator-activated receptor (PPAR) and has the ability to regulate serum triglyceride levels. This ability is related to increased lipoprotein lipase (LPL) activity, promoting lipolysis in AT, and improving glucose tolerance (Ingerslev et al., 2017). Specifically, plasma ANGPTL4 acts on myotubes, and is well-established as a myokine derived from physical exercise (exercise factor; Raschke and Eckel, 2013; Ingerslev et al., 2017). The performance of this protein is related to several physiological processes, among them are insulin sensitivity, lipid metabolism, adipogenesis (Cinkajzlova et al., 2018), and the increase of circulating fatty acids, which are generated mainly by chronic caloric restrictions, fasting, and aerobic physical training (Raschke and Eckel, 2013).

It was seen that when muscle contraction was stimulated in myocytes during exercise using electrical pulse stimulus, there was an increase in the gene expression of ANGPTL4 after 4 h, and an increase of the supernatant proteins after 8 h of stimulation, corroborating the link between exercise and the expression of ANGPTL4 (Scheler et al., 2013). However, there are contrary findings, as in the study by Catoire et al. using acute endurance exercise in a single leg, ANGPTL4 was shown to be more highly expressed in unexercised legs than in exercised legs (Catoire et al., 2014).

The practice of fasting combined with physical exercise is indicated as an important inducer of ANGPTL4 expression and may be beneficial to those with high serum levels of long chain fatty acids (Ingerslev et al., 2017). In addition, an increase in serum ANGPTL4 in obese patients (with or without T2DM) was observed, and the same was observed in patients who underwent bariatric surgery and in fasting patients, while it was reduced in patients with anorexia (Cinkajzlova et al., 2018). However, even with published data indicating a role for ANGPTL4 in obesity, and glucose intolerance (Morris, 2018), this protein is still poorly studied and there is much about its therapeutic role that needs to be further investigated.

#### BAIBA

β-aminoisobutyric acid (BAIBA) are natural metabolites of thymine and valine secreted primarily by myocytes during the practice of physical exercise (Roberts et al., 2014). The increase of PGC1α observed during physical exercise results in a concomitant increase in BAIBA serum levels. In the same study, *in vitro*, when BAIBA was cultivated with primary cells from subcutaneous adipose tissue (scAT), there was an up regulation of brown and beige adipocytes marker, such as UCP1 and CIDEA. In this way, it was shown that BAIBA has a potential to induce browning in white adipocytes, in addition to demonstrating that physical exercise increases its production by the body. Moreover, BAIBA has been proposed as a promising molecule capable of inducing the thermogenic framework by an independent pathway of adrenergic activation (Jeremic et al., 2017).

BAIBA also has an important therapeutic role against obesity (Ginter and Simko, 2014), mainly by stimulating the oxidation of fatty acids, reducing the process of lipogenesis in WAT, and attenuating inflammation and insulin resistance (Jung et al., 2018). The regulation of BAIBA is mediated through AMPK signaling, and plays an important role in several pathologies. In addition to obesity, BAIBA is reported to attenuate hepatic apoptosis by reducing ER stress on hyperlipidemia and improving renal fibrosis (Jung et al., 2018). Furthermore, it has been shown that circulating levels of BAIBA have an important role against cardiometabolic risks (Kammoun and Febbraio, 2014). Thus, with these data, it is possible to determine that the crosstalk between SkM, cardiac muscle, liver and WAT and BAT has a strong correlation with the practice of physical exercise (Kammoun and Febbraio, 2014).

#### Myonectin

Myonectin, also known as C1q/TNF-related protein 15 (CTRP15), is a new myokine recently found as a protein typical of fatty acid metabolism in response to exercise (Seldin et al., 2012; Toloza et al., 2018), although in other studies the relationship between the expression of myonectin and physical exercise is contradictory (Peterson et al., 2014).

The study by Seldin et al., which made a general characterization of this myokine, showed that it is widely expressed in SkM, more precisely in myotubes (Seldin et al., 2012). In addition, it is described as a potent inducer of cell differentiation in C2C12 cells, which, according to the authors, indicates that this protein is produced in muscle fibers, not in satellite cells (Seldin et al., 2012). A test performed *in vivo* using the soleus and plantaris muscles showed increased gene expression and circulating levels of myonectin in animals that had been fed (compared to animals that had not). It was also shown that in the presence of carbohydrates or lipids in the gastrointestinal tract there was potent stimulation of myonectin expression in the assessed muscles (Seldin et al., 2012), suggesting a strong correlation between myonectin and nutrient uptake (Toloza et al., 2018).

High levels of myonectin were observed in individuals with T2DM, glucose intolerance, and obesity (Li et al., 2018). Other members of the CTRP family are expressed by AT, and they are described as adiponectin paralogue genes (Wong et al., 2008). This gene family is up-regulated in ob/ob mice and are highly expressed when stimulated with peroxisome proliferator-activated receptor gamma (PPARγ) agonists (Wong et al., 2008), however, because it is newly discovered, there is as yet little in the literature regarding the direct crosstalk between myonectin and AT.

In summary, myonectin, as well as irisin, promote glucose and fatty acid uptake and oxidation in both the liver and AT, acting more specifically on lipid metabolism mediated by CD36, fatty acid transporter protein (FATP) and fatty acid binding protein (FABP4), but not participating in lipolysis and glucose homeostasis (Gamas et al., 2015). Myonectin, is, therefore, a potent target for studies that focus on identifying future therapies, mainly related to insulin resistance. It is fundamental to characterize molecules that may present a function mimicking the effects of physical exercise and consequently positively modulating lipid homeostasis and lipogenesis in AT (Gamas et al., 2015).

## Conclusion

Over the last decades, exercise training has been suggested as a preventive and therapeutic strategy for managing and treating several NCDs, including T2DM, hypertension, heart disease, obesity, and sarcopenia. Physical exercise is known to improve metabolic health through adaptations to several tissues, including SkM and AT. Although metabolic improvements from exercise training in SkM and AT are important in themselves, an important concept that has been evidenced in exercise physiology is the concept of tissue communication, or “crosstalk.” In addition, many studies have proposed that in NCDs, mainly in those related to metabolic diseases such as obesity, a low-grade chronic inflammatory profile is a well-characterized scenario. Interestingly, several recent studies have characterized and identified various myokines released from SkM during and after a single bout of exercise. In particular, myokines may act pleiotropically, mediating many aspects related to AT metabolism. Myokines have also provided a new basis to understand the molecular mechanisms underlying the beneficial effects of exercise training on the reduction of morbidity and mortality rates. Although the identified myokines share a common role in regulating metabolism, how each myokine works and how these myokines work together still remains to be elucidated. In addition, given the role of myokines in fine-tuning the metabolic process associated with exercise, the development of specific exercise regimens or compounds derived from myokines that mimic the effects of exercise are promising areas to explore in the treatment of metabolic diseases through exercise.

## Author contributions

MB, LL, and ML conceived the review. LL and ML analyzed the data and MB, LL, and ML wrote the paper.

### Conflict of interest statement

The authors declare that the research was conducted in the absence of any commercial or financial relationships that could be construed as a potential conflict of interest.
